# Natural Fiber-Reinforced Polylactic Acid, Polylactic Acid Blends and Their Composites for Advanced Applications

**DOI:** 10.3390/polym14010202

**Published:** 2022-01-05

**Authors:** R. A. Ilyas, M. Y. M. Zuhri, H. A. Aisyah, M. R. M. Asyraf, S. A. Hassan, E. S. Zainudin, S. M. Sapuan, S. Sharma, S. P. Bangar, R. Jumaidin, Y. Nawab, A. A. M. Faudzi, H. Abral, M. Asrofi, E. Syafri, N. H. Sari

**Affiliations:** 1 School of Chemical and Energy Engineering, Faculty of Engineering, Universiti Teknologi Malaysia (UTM), Johor Bahru 81310, Malaysia; 2 Centre for Advanced Composite Materials (CACM), Universiti Teknologi Malaysia (UTM), Johor Bahru 81310, Malaysia; shukur@utm.my; 3 Institute of Tropical Forestry and Forest Products, Universiti Putra Malaysia, Serdang 43400, Malaysia; aisyah.humaira@upm.edu.my (H.A.A.); edisyam@upm.edu.my (E.S.Z.); sapuan@upm.edu.my (S.M.S.); 4 Advanced Engineering Materials and Composites Research Centre (AEMC), Department of Mechanical and Manufacturing Engineering, Universiti Putra Malaysia, Serdang 43400, Malaysia; 5 Institute of Energy Infrastructure, Universiti Tenaga Nasional, Jalan Ikram-Uniten, Kajang 43000, Malaysia; asyrafriz96@gmail.com; 6 Department of Mechanical Engineering, IK Gujral Punjab Technical University, Punjab 144603, India; shubham543sharma@gmail.com; 7 Department of Mechanical Engineering, University Centre for Research and Development and Chandigarh Universiti, Pubjab 140413, India; 8 Department of Food, Nutrition and Packaging Sciences, Clemson University, Clemson, SC 29631, USA; snehpunia69@gmail.com; 9 Fakulti Teknologi Kejuruteraan Mekanikal dan Pembuatan, Universiti Teknikal Malaysia Melaka, Jalan Hang Tuah Jaya, Durian Tunggal, Melaka 76100, Malaysia; ridhwan@utem.edu.my; 10 Textile Composite Materials Research Group, National Center for Composite Materials, Faculty of Engineering and Technology, National Textile University, Faisalabad 37610, Pakistan; yasir.nawab@yahoo.com; 11 School of Electrical Engineering, Universiti Teknologi Malaysia, Johor Bahru 81310, Malaysia; athif@utm.my; 12 Department of Mechanical Engineering, Andalas University, Padang 25163, Indonesia; habral@yahoo.com; 13 Department of Mechanical Engineering, University of Jember, Kampus Tegalboto, Jember 68121, Indonesia; asrofi.teknik@unej.ac.id; 14 Department of Agricultural Technology, Agricultural Polytechnic, Payakumbuh 26271, Indonesia; edisyafri11@gmail.com; 15 Mechanical Engineering Department, Faculty of Engineering, University of Mataram, Mataram 83115, Indonesia; n.herlinasari@unram.ac.id

**Keywords:** natural fiber, polylactic acid, polylactic acid blends, polylactic acid composites

## Abstract

Polylactic acid (PLA) is a thermoplastic polymer produced from lactic acid that has been chiefly utilized in biodegradable material and as a composite matrix material. PLA is a prominent biomaterial that is widely used to replace traditional petrochemical-based polymers in various applications owing environmental concerns. Green composites have gained greater attention as ecological consciousness has grown since they have the potential to be more appealing than conventional petroleum-based composites, which are toxic and nonbiodegradable. PLA-based composites with natural fiber have been extensively utilized in a variety of applications, from packaging to medicine, due to their biodegradable, recyclable, high mechanical strength, low toxicity, good barrier properties, friendly processing, and excellent characteristics. A summary of natural fibers, green composites, and PLA, along with their respective properties, classification, functionality, and different processing methods, are discussed to discover the natural fiber-reinforced PLA composite material development for a wide range of applications. This work also emphasizes the research and properties of PLA-based green composites, PLA blend composites, and PLA hybrid composites over the past few years. PLA’s potential as a strong material in engineering applications areas is addressed. This review also covers issues, challenges, opportunities, and perspectives in developing and characterizing PLA-based green composites.

## 1. Introduction

Despite the fact that fiber-reinforced polymers (FRPs) have been used in a wide range of engineering applications, especially where high strength and stiffness are needed, conventional FRP composites often pose significant challenges in terms of reuse or recycling at the end of their useful lives, owing to the nonbiodegradable fibers and matrixes. As a result, green composites made of natural fibers and biodegradable polymers have been created. Major oil shortages due to the limited existence of fossil resources, an increase in the release of greenhouse gases into the environment as a result of burning fossil resources, and a significant increase in the amount of composite waste have all led to a new public understanding of green composites [1,2,3]. Natural fibers do not have these drawbacks because they are produced from the natural process of photosynthesis, which involves CO_2_ absorption and the release of O_2_ into the atmosphere. They even decompose naturally, which means they do not pollute the environment [4].

In the 1980s, researchers began developing partially biodegradable composites made of cellulosic fibers and thermoset resin. It was documented in the 1990s that thermoplastic resin was used to fabricate wood flour (WF)-reinforced composites. At present, using different natural fibers (e.g., flax, ramie, hemp, etc.) and biodegradable polymers (e.g., starch, cellulose, or vegetable oil derivatives), a number of partially biodegradable and green composites with reasonably good mechanical properties have been created [5]. The field of biodegradable material application is constantly expanding because of their improving properties, which in many cases mimic petrochemical polymers [6]. A passenger bus’s middle section between the headlights above the fender is an example. The part is made from hemp fiber bundles and PTP or prepolymer made of triglycerides and polycarbocxylic acids, a thermosetting resin based on vegetable oil, and is made using the sheet molding compound technique (SMC) [7,8].

The properties of the natural fibers used as reinforcement determine the efficiency of green composites [9,10]. Rather than having disadvantages such as low modulus of elasticity, high moisture absorption, and decomposition in the biological assault, the most significant characteristic of green composites is their complete biodegradability with no negative environmental impact [11,12]. Further feasibility research into the possible use of natural fibers as reinforcing compounding materials for use in many applications is also generating many economic and environmental impacts [7,13,14,15,16,17,18,19]. The current study examines recent research and developments in PLA-based green composites and their mechanical properties in terms of tensile strength, compressive strength, flexural properties, and impact strength, as well as some of the fundamental issues in the construction of such composites.

## 2. Natural Fiber

Cellulose is a natural polymer that has a high strength and stiffness per weight and is used to build long, fibrous cells. The stem, leaves, and seeds of plants all contain these cells. Plant, animal, and mineral fibers are the three general types of natural fibers. There are several subcategories under these three groups, such as leaf, silk, and asbestos. Wood fibers from trees are the most abundant of these natural fibers. In this subtopic, a more detailed overview regarding constituents, chemical composition, and mechanical properties is presented.

### 2.1. Constituents and Types of Green Composites

**Figure 1:**
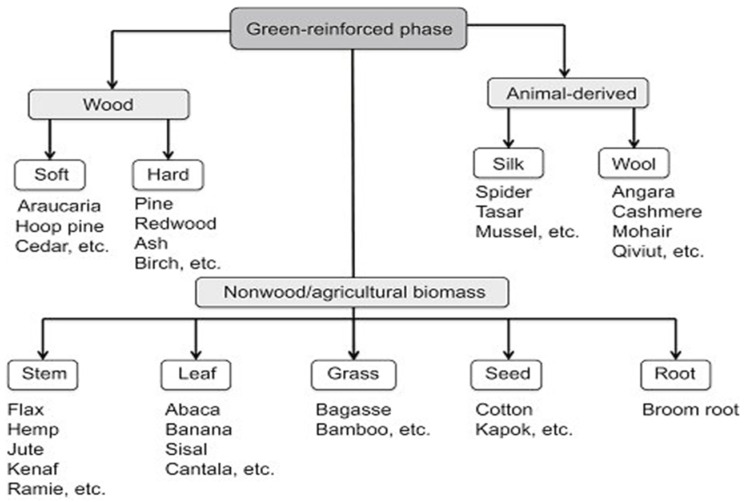
The natural reinforcement constituents of green composites. (Reproduced with a copyright permission from Nagalakshmaiah et al. [23]).

**Figure 2:**
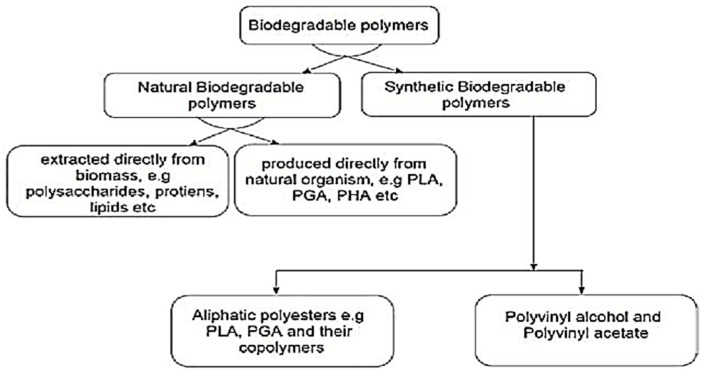
The biodegradable classification of polymers constituents in green composites. (Reproduced with a copyright permission from Karande et al. [24]).

Green composites consist of the matrix phase and reinforcement materials as the main constituents, and the characteristics of green composites are determined mainly by the interface conditions [20,21]. In defining the overall properties of the green composite, the reinforced phase plays an important role [22]. The matrix isolates the fibers to avoid abrasion and the development of new surface flaws besides functioning as a bridge that holds the fibers intact. Several factors that express a good matrix are the ability of the matrix to deform easily under applied load, transferring the load onto the fibers, as well as distributing the stress concentration evenly. Examples of matrix materials are propylene (PP), polyethylene (PE), and epoxy, also known as petroleum-derived non-biodegradable polymers. Normally, with the addition of reinforcements (the second main component) to the matrix, the mechanical properties of the neat resin system are greatly improved. Hence, the different constituents in the intermixed state create an interface between matrix and green fibers called the contiguous region. In some cases, an added phase or coating is present in the contiguous region, improving surface-wetting characterization, i.e., matrix reinforcement interphase. Furthermore, the primary element affecting the green composite properties is the interface adhesive conditions between natural fibers and matrixes. Surface cleaning methods such as silane, alkaline, and acetone treatment are used to improve wettability with the polymer matrix and reduce the development of voids. Figure 1 and Figure 2 show the constituents of the reinforcements and the polymer matrix of green composites, respectively.

Natural fibers reinforced with a petroleum-based nonbiodegradable polymer matrix produce partially biodegradable composite, and natural fibers reinforced with biodegradable resin matrix produce green or fully biodegradable composite. The addition of two different natural fibers into the polymer matrix results in “hybrid” green composites [25,26,27,28,29,30]. Green composites can be classified depending on the nature of reinforcements used and their functional behavior. Based on the nature of reinforcements, they can be further classified as unidirectional and bi-directional continuous fiber green or discontinuous reinforcement composites. As for the functional behavior, it is divided into functionally graded and smart green composites.

### 2.2. Chemical Composition of Green Fibers

Environmental factors such as growth, location, nutrition, temperature, season, and local climatic condition influence the properties and quality of natural fibers [4,31]. Furthermore, the plant part, retting process, variety, and harvesting conditions also have a role in the differences in their characteristics [32,33]. Wood is a three-dimensional polymeric aggregate made up mostly of cellulose, hemicelluloses, and lignin. Plant fibers have the same constituent of chemical structure but with different compositions, which makes the fibers behave differently [34,35,36].

All plant fibers are mainly made up of cellulose, which is the most basic structural component [37,38,39,40]. It is a kind of natural polymer. Cellulose molecules are made up of glucose units linked together in long chains (β-1,4 glycoside linkages bind the repeating units of D-anhydro glucose C_6_H_10_O_5_), linked together in microfibrils. The hydrogen bonding in cellulose determines its crystallinity, which controls the physical properties of natural fibers. This is the primary component that gives them resilience, stiffness, and stability. Polysaccharides bound together in short, branching chains known as hemicelluloses [41]. They are closely related to cellulose microfibrils and help to embed the cellulose in a matrix. Hemicelluloses are naturally hydrophilic in addition to their molecules having a lower molecular weight than cellulose. Lignin is a polymer made up of complex aromatic hydrocarbons that give plants their rigidity. Plants could not grow to great heights without lignin. Lignin is a less polar three-dimensional polymer with an amorphous structure and a high molecular weight than cellulose. Within and between fibers, it serves as a chemical adhesive and provides cell walls with reinforced mechanical strength and hydrophobicity [42,43]. Table 1 below shows the chemical composition of popular plant fibers.

### 2.3. Mechanical Properties of Green Fibers

Green fibers have good thermal and acoustic insulation properties due to their hollow and lignocellulosic structure in nature. Despite the mechanical properties of green fibers being comparatively lower than the synthetic fibers, it can be enhanced by proper surface treatment of fibers. In addition, industries are fond of green fibers because of their low densities, low cost, and high specific modulus. In comparison, based on Table 2, Young’s modulus of glass fibers is almost the same as green fibers. In contrast, the tensile strength of glass fibers is higher than the plant fibers and vice versa for the specific modulus. Therefore, the aforementioned properties make natural fibers a suitable candidate for green composites application.

## 3. Polylactic Acid (PLA)

**Figure 3:**
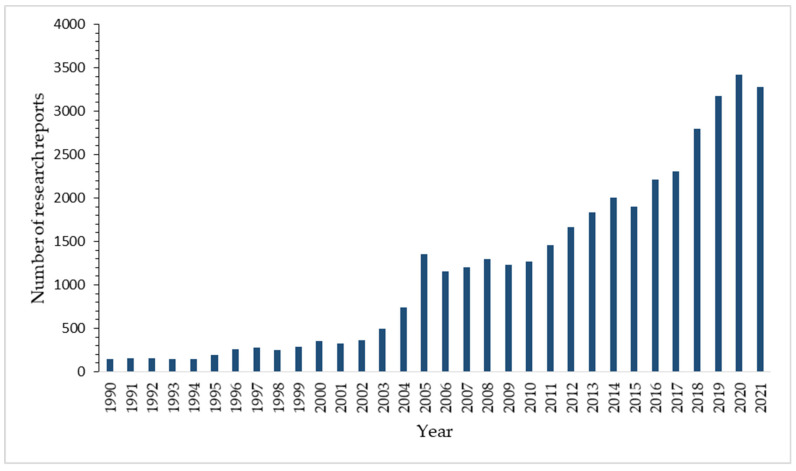
Number of research reports published since 1990 based on the Scopus search.

**Figure 4:**
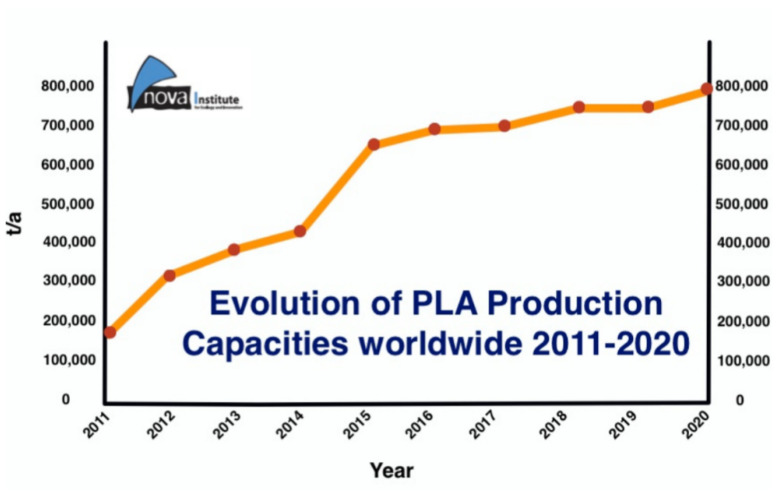
Global PLA market forecast, 2011−2020. Source: Nova Institute, [62].

Polylactic acid (PLA) is a renewable thermoplastic polymer matrix and an aliphatic polyester made from lactic acid and has been a key component in producing biodegradable products, making it one of the most promising biopolymers used in the world. Generally, PLA is produced from agricultural source materials that have been fermented to produce lactic acid [55,60,61]. The cyclic dimer (lactide) of lactic acid is polymerized through the combination of oligomerization and cyclic dilactone processes. PLA has been proven to have exceptional properties through thorough studies since 1990 (Figure 3) and was estimated to increase production capacities (Figure 4). Because PLA is completely biodegradable, it has been commercialized for applications that need biodegradability, such as disposable cups and plates, packaging materials, disposable cutlery, and plastic bags.

### 3.1. Advantages and Disadvantages

PLA offers a number of benefits over the other biopolymers, including environmentally friendly, i.e., biodegradable, compostable, and recyclable. PLA production consumes carbon dioxide, and it is derived from renewable resources such as sugarcane, corn, potatoes, cassava, wheat, and rice [63,64]. In a few months to a few years, the degradation of PLA occurs by hydrolysis lactic acid to water, carbon monoxide, and humus, which is metabolized by microbes [65]. Urayama et al. [66] discovered that after 20 months in soil, PLA plates decrease their molecular weight by 20%. According to Teixeira et al. [67], the PLA degradation is affected not only by the characteristics of the specimen, such as degree of crystallinity, molecular weight, sample morphology, and molecular structures, but also by the surrounding environmental conditions. In addition, the surface of PLA is very permeable. As a result, microorganisms in nature may readily enter, assisting in the rapid natural breakdown of PLA. Furthermore, PLA has the ability to recycle back to lactic acid via hydrolysis or alcoholysis, hence making PLA an attractive biopolymer option.

PLA is a biocompatible material that is safe for use in applications such as food containers and medical devices. PLA does not produce toxic nor carcinogenic effects that impact the local tissues, and the degradation of material should not disrupt the healing process of the tissues [68]. Thus, PLA is biocompatible and suitable for biomedical applications. PLA is broken down into α-hydroxy acid when implanted in living organisms and is then merged into the tricarboxylic acid cycle before being excreted [68,69]. In addition, at the lower composition of PLA, it degrades non-toxically, making it a natural choice for biomedical applications [68,70,71]. The Food and Drug Administration (FDA) has also approved PLA for direct contact with biological fluids [72,73].

PLA also has good processability where PLA can be processed through many processing possibilities such as injection molding [74,75], film extrusion [76,77], thermoforming [78], extrusion blow molding [79], injection blow molding [80,81], injection stretch blow molding [82], film forming, cast film extrusion [83], and fiber spinning [84]. The thermal processibility of PLA is better than that of other biopolymers compared to other polymers such as poly(hydroxyalkanoates)(PHAs) and polyethylene glycol (PEG) [82].

Additionally, the processing advantages of PLA can provide significant energy as well as cost savings due to its lower crystallizing, drying, and melt-processing temperatures. Vink et al. [63] mentioned the lower energy requirement to produce PLA, i.e., 25–55% less than that of petroleum-based polymers, and it is predicted that this energy-saving can further be decreased by another 10% in the future. Hence, PLA production is also quoted to be a cheap alternative as well due to its low energy production.

There are, however, some disadvantages of using PLA over other materials, including poor toughness. Despite having tensile strength and elastic modulus that are comparable to polyethylene terephthalate (PET) [82], PLA has less than 10% elongation at break, making it a very brittle material [85]. Therefore, PLA is unsuitable for applications requiring plastic deformation at higher stress levels [86].

The rate of degradation of PLA is very slow in ambient temperatures. Several factors affect the degradation rate of PLA: crystallinity, molecular weight, morphology, molecular weight distribution, stereoisomeric content, and water diffusion rate into the polymer [87,88]. The degradation rate of PLA causes a serious setback in consumer commodities disposal. However, PLA can be degraded by several processes such as hydrolysis, thermal degradation or photodegradation, by applying UV radiation.

PLA is a relatively hydrophobic material, has low water absorption, and its ester bond is less labile to hydrolysis due to the methyl group’s steric hindrance. Due to the nature of PLA being hydrophobic, this results in low cell affinity, which in return causes an inflammatory response from the living host when having direct contact with biological fluids [89]. PLA also lacks reactive side-chain groups. It is a chemically inert polymer with insufficient reactive side-chain groups, making surface and bulk changes difficult. Surface and bulk modifications of PLA are challenging because it is chemically inert and has no reactive side-chain groups [90,91].

### 3.2. Physical Properties of PLA

In general, dioxane, acetonitrile, chloroform, methylene chloride, 1,1,2-trichloroethane, and dichloroacetic acid can dissolve PLA and most of its derivatives. At low temperatures, PLA products can be partially dissolved in ethyl benzene, toluene, acetone, and tetrahydrofuran (THF). However, at boiling temperatures, they can be fully dissolved in the aforementioned solvents. Water, alcohol, and aliphatic hydrocarbons such as hexane and heptane, which can be used as antisolvents, are incompatible with PLA products. In acetone, ethyl acetate, or THF solvents, PLA with high crystallinity and molecular weight is insoluble, and only polymers with lower molecular weights can be dissolved.

Polymers can be semicrystalline or amorphous. Semicrystalline polymers have regular repeating units that cause the chains to fold into crystallites, which are dense regions. The PLA can be either amorphous or semicrystalline at room temperature, depending on the molecular weight and stereochemistry structure. It has been reported that the melting enthalpy for 100% crystallinity PLA is 93–148 J/g [92]. PLA is made of pure L-lactic and D-lactic isomers, generating poly-l-lactic acid (PLLA) and poly-d-lactic acid (PDLA) homopolymers. Additionally, Poly-d,l-lactic acid (PDLLA) copolymer is produced by using a racemic combination of L- and D-monomers. It was reported that PDLLA is an amorphous polymer with no melting point, while PLLA is a semi-crystalline polymer. Because of the existence of crystalline areas, the degradation rate of PLLA is considerably slower than that of PDLLA [93].

The amorphous and crystalline densities of PLLA have been stated to be 1.248 g/mL and 1.290 g/mL, respectively. L-lactide has a density of 1.36 g/cm^3^, o-lactide has a density of 1.33 g/cm^3^, crystalline polylactide has a density of 1.36 g/cm^3^, and amorphous polylactide has a density of 1.25 g/cm^3^. The viscosity of polylactic acid solutions increases as the concentration increases but decreases as the temperature rises.

The distribution functions of crystallites in PLA were determined as a function of spiral angle by Kobayashi et al. [94]. They also verified the helical structure of PLLA and PDLA and showed that PLLA had developed tremendous optical activity in the solid-state. In the lower UV-C range (190–220 nm), PLA has almost no UV transmission. The amount of UV light transmitted by PLA is substantially increased at 225 nm. The overall properties of PLA can be regulated by using special catalysts that have isotactic and syndiotactic content with different enantiomeric units [95]. Table 3 summarizes the general properties of commercial amorphous PLA.

### 3.3. Mechanical Properties of PLA

PLA is a bio-based polymer with the potential to replace petroleum-based plastics due to its stiffness and strength. The mechanical characteristics of PLA may range from elastic soft to stiff, high-strength materials, depending on numerous factors such as polymer structure, material formulation, orientation, crystallinity, and molecular weight as shon in Table 4. When higher mechanical properties are needed, semicrystalline PLA is favored over amorphous PLA. Farah et al. [91] discovered that generally, PLA has a tensile modulus of 3–4 GPa, a tensile strength of 50–70 MPa, a flexural strength of 90–100 MPa, and an elongation at break of around 1–4%.

Unlike its thermal properties, PLA’s mechanical properties and crystallization behavior are highly dependent on Mw and the stereochemical make-up backbone [99]. For instance, when the Mw is increased from 50 to 100 kDa [100], the tensile modulus of PLLA increases by a factor of two, and tensile strengths of 15.5, 80, and 150 MPa were obtained for varying the Mw from 50 to 150 to 200 kDa, respectively [101].

Tensile properties—tensile strength (σ, in MPa), tensile modulus (E, in GPa), ultimate strain (ε, in percent), and polymer density (p, in g/cm^3^)—are the mechanical properties of PLA that have been studied the most in relation to a collection of biopolymers. Tensile properties are clearly best for the densest recorded polymers, especially poly (glycolic acid) (PGA). PCL, on the other hand, appears to be the most fragile polymer, with a heavy strain at failure. Data on flexural properties were insufficient to make a comparison. However, since flexural and tensile properties are largely associated, the tendencies observed here are likely to be the same as those observed when comparing flexural properties [101].

PLA tensile strength and modulus ranges are compared to various biopolymers. These characteristics are crucial since they specify the dimensions required for a given mechanical strength or stiffness. PGA and PLLA appear to be the best options in this situation, despite their lack of utility as a composite matrix, while PCL and polyhydroxybutyrate (PHB, σ*8.8 MPa and E*7.8 MPa) are obviously the worst [101].

In terms of flexural properties, PLA is a promising thermoplastic polymer having a flexural strength of up to 140 MPa and Young’s modulus of 5–10 GPa and good optical characteristics. The addition of natural fibers to PLA may enhance its mechanical characteristics. The applicability of natural fibers such as hemp and flax as a reinforcement material was addressed by Loos et al. [102], and the mechanical characteristics of flax and hemp employed in polymer reinforcement were studied. Previously, researchers showed that adding natural fibers to PLA bio composites improves their flexural, tensile, and impact strength [103,104,105,106,107].

**Table 4:** Mechanical properties of pure PLA.

Mechanical Test	Parameter and Values	Ultimate Tensile Strength (MPa)	Elastic Modulus (MPa)	Ref.
Tensile	Layer height (0.2 mm)	60.4	3480	[108]
	Raster angle (0/90, 45/45)	54.9	3336	
Tensile	Infill percentage (60%, 100%)	62.5	-	[109]
	Build orientation (front, side)	57.0	-	
	Layer height (0.15, 0.4 mm)	57.0	-	
Tensile	Raster angle (0, 45, 90)	64.03	3600	[110]
Tensile	Raster angle (0, 45, 90)	38.65	1538	[111]
Tensile	Raster angle (0, 45, 90)	38.70	1538	[112]
Tensile	Layer thickness (0.1, 0.12, 0.15, 0.18, 0.2 mm)	49.29	3497.63	[113]
	Raster angle (0, 18, 45, 72, 90)	53.59	3388.57	
	Number of shells (2, 3, 4, 5, 6)	50.67	3189.01	
Tensile	Layer height (0.1 mm)	45.56	1125	[114]
Compression	Raster angle (0/90, 45/135, 0/45/90/135)	-	408–1018	[115]
Tensile	Layer thickness (0.06, 0.12,0.18, 0.24 mm) Build orientation (upright, flat, on-edge) Feed rate (20, 50, 80 mm/s)	89.1	4409	[116]
Tensile	Build orientation (front, side)	66.96	1350	[117]
Tensile (quasi-static loading)	Raster angle (0, 30, 45, 60, 90)	45.8	3372	[118]

### 3.4. Thermal Properties of PLA

**Figure 5:**
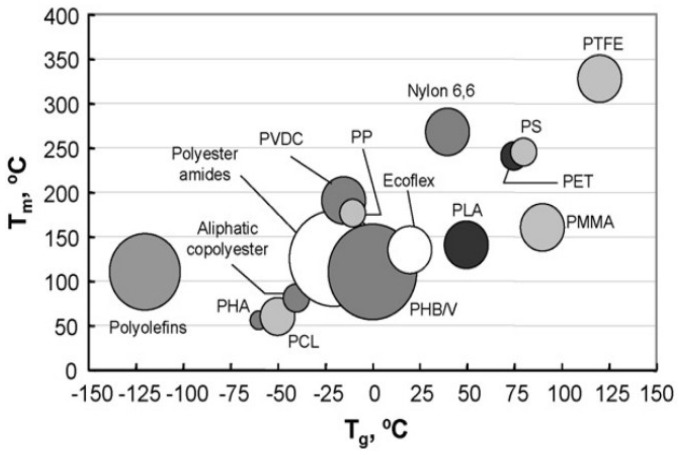
Glass transition and melting temperature of PLA with other thermoplastics. (Reproduced with a copyright permission from Tsuji and Ikada [95]).

T_g_ and T_m_ are present in semicrystalline PLA, as they are in many thermoplastic polymers. PLA is rubbery above T_g_ (58 °C), but it becomes a glass below T_g_, which can also creep until cooled to its transition temperature of approximately 45 °C, below which it acts as a brittle polymer [119]. PLA’s T_g_ and T_m_ values are compared to those of other polymers in Figure 5; PLA has a relatively high T_g_ and low T_m_ as compared to other thermoplastics, as seen.

**Figure 6:**
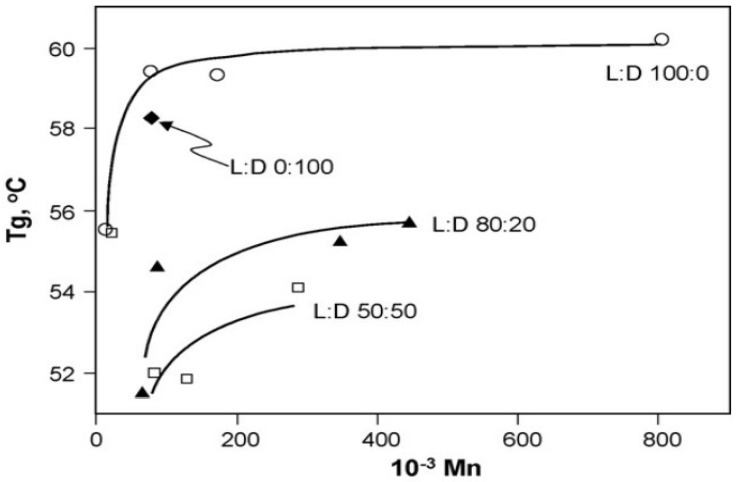
Glass transition temperature for PLAs of different L-contents as a function of molecular weight (Reproduced with a copyright permission from Naser et al. [123]).

PLA’s T_g_ is determined by the molecular weight and optical purity of the polymer (Figure 6). The T_g_ increases with molecular weight, reaching maximum values of 60.2, 56.4, and 54.6 °C for PLA with 100%, 80%, and 50% l-stereoisomer contents, respectively, at the infinite molecular weight. PLA with a higher l-lactide content has higher T_g_ values than PLA with the same d-lactide content [120]. Tsuji and Ikada [95] found similar relationships. In addition, PLA characteristics may be adjusted by using isotactic and syndiotactic content catalysts with various enantiomeric units. For instance, PLA with a PLLA concentration of more than 90% is crystalline, while PLA with a lower optical purity is amorphous. With decreasing quantities of PLLA, the T_m_ and T_g_ of PLA drop [121,122]. Table 5 displays the glass transition and melting temperatures of various PLA polymers made with different copolymer ratios.

In general, the relationship between T_g_ and molecular weight can be represented by the Flory–Fox equation:

T_g_ = (T^∞^ − K)/M_n_ (1)

where T^∞^ is the T_g_ at the infinite molecular weight, K is a constant representing the excess free volume of the end groups for polymer chains, and M_n_ is the number average molecular weight. The values of T^∞^ and K are around 57–58 °C and (5.5–7.3) × 10^4^ as reported in the literature for PLLA and PDLLA, respectively [124].

PLA’s glass transition behavior is also influenced by the polymer’s thermal background. The polymer would be extremely amorphous if it is quenched from the melt at a high cooling rate (>500 °C/min, as in injection molding). Under atmospheric conditions, PLA polymers with low crystallinity have a propensity to age rapidly in a matter of days [125,126].

PLA’s T_m_ is also determined by its optical purity. For stereochemical pure PLA, the highest practical obtainable T_m_ is about 180 °C, with an enthalpy of 40–50 J/g. The following expression can be used to estimate the relationship between Tm and meso-lactide content:

T_m_(°C) ≈ 175 °C−300 W_m_ (2)

where W_m_ is the fraction of meso-lactide below 0.18 level, and 175 °C is the melting temperature of PLA made of 100% l-lactide. Typical T_m_ values for PLA are in the range of 130–160 °C. The T_m_ depression effect of meso-lactide has several important implications as it helps expand the process windows, reduce thermal and hydrolytic degradation, and decrease lactide formation. The heat capacity of PLA in solid and liquid states ranging from 5 to 600 K. The heat capacity (C_p-liquid_, JK^−1^ mol^−1^) can be represented in a simple form:

C_p-liquid_ = 120.17 + 0.076T (3)

where T is in Kelvin (K).

## 4. Processing of PLA Green Composites

The majority of green composites are made using the same processes as conventional synthetic FRP matrix composites, divided into an open and closed mold. The fabrication methods and processing parameters for PLA-based green composites are summarized in Table 6. Open mold processes include hand layup, spray up, tape layup, filament winding, and the autoclave system. Closed mold processes include compression molding, injection molding, and transfer molding. Table 6 indicates that the researchers used hot pressing, wet impregnation method, twin-screw extrusion and injection molding, and two-roll plastics mill and hot pressing.

Carding, treatment with a 3-glycidoxypropyl trimethoxy silane, and hot pressing were used to fabricate kenaf fiber-reinforced polylactide biocomposites by Lee et al. [127]. Carding was used to create a uniform mixture of the two fibers, which was then followed by needle punching, pre-pressing, and eventually hot pressing to create the composite material. The mat’s thickness was reduced by pressing the PLA/kenaf nonwoven web formed after the carding process. The silane coupling agent was applied to the pre-pressed nonwoven web in quantities of 1, 3, and 5 parts per hundred (pph) of the pre-pressed composite material. For two hours, the silane was allowed to penetrate and respond with the pre-pressed mat. Finally, the silane-treated pre-pressed mat was hot-pressed at 200 °C for 5 min at 0.7 MPa pressure. Using a film-stacking technique, David Plackett et al. [128] created a PLA/jute biodegradable composite containing around 40% jute fiber by weight. Using a single-screw extruder, PLA was first converted into a 0.2 mm thick film for this analysis. Sections of jute fiber mats were stacked with multiple PLA film layers on either side inside a metal frame to create layups. At the top and bottom of the frame, Teflon sheets were used. The layups underwent a rapid press consolidation process that included (a) precompression, (b) touch heating under vacuum, (c) rapid transfer to a press for consolidating and cooling, and (d) removal of the finished portion from the press [129].

**Table 6:** Fabrication methods and processing parameters for PLA-based green composites.

Fiber	Process	Temperature	Pressure	Time of Heating	Reference
Kenaf	Wet impregnation method	Room temperature	Process under vacuum	No heating, 24 h drying	[130]
Kenaf	Hot pressing	160 °C	10 MPa	10 min	[131]
Flax	Twin screw extrusion + injection molding	250 °C	70 MPa Screw speed—250 rpm	-	[132]
Chicken feather	Twin screw extrusion + injection molding	180 °C	Screw speed—100 r/min	10 min	[133]
Bamboo	Twin-screw extrusion + injection molding	180 °C	50–60 MPa	-	[134]
Bamboo	Compounding + injection molding	170 °C	Screw speed—150 r/min	-	[135]
Treated ramie	Two-roll plastics mill + hot pressing	140–170 °C	20 MPa	4 min (hot press)	[136]
Short ramie	Two-roll plastics mill + hot pressing	140–170 °C	5 MPa	4 min (hot press)	[137]
Ramie and jute	Two-roll plastics mill + hot pressing	140–170 °C	20 MPa (of hot press)	4 min (hot press)	[137]

## 5. PLA-Based Green Composites

The increased use of petroleum-based polymer composites has increased the environmental “burden”. Increasing public consciousness of environmental issues has compelled manufacturers and companies to seek out more environmentally friendly materials for their goods. Composites based on natural fibers with polypropylene as a matrix material, for example, are now widely used in automotive applications [138]. A number of studies have been conducted on composites made with renewable polymer matrices derived from plants, such as PLA, cellulose esters, polyhydroxyalkonoates (PHAs), and so on [139,140,141,142,143,144]. As a result, research and production of biopolymer-based natural fiber composites have piqued the interest of materials scientists and engineers from both a scientific and environmental standpoint.

Biodegradable polymers can be reinforced with natural fibers to create environmentally friendly and biodegradable hybrid composites [145]. Bio-composites are biodegradable, reusable, and renewable, reducing reliance on finite petroleum resources while also reducing environmental impact [146]. PLA’s excellent mechanical properties and barrier capability can be used to create biomaterials that are ideal for a wide range of applications [147]. The drawbacks of PLA include its inherent brittleness, water sensitivity, and low impact strength [129]. These issues can be mitigated by adding fibers and/or fillers, which is a simple way to boost the polymer’s overall properties [148,149,150,151].

### 5.1. Mechanical Properties of PLA-Based Green Composites

Mechanical properties among the most critical factors in composite characterization as they determine the final strength of the products. Various characterization techniques have been used to investigate the influence of different fibers on the behavior of the resulting composite material. Fiber volume fraction, stacking sequence of the fiber layers, processing methods, fiber treatment, and the impact of environmental conditions, i.e., hydrothermal ageing, moisture sorption, and chemical resistance, are all factors considered in mechanical characterization [152,153,154,155]. Natural fibers such as hemp, ramie, kenaf, rice straw, abaca, wood, coir, jute, sisal, bamboo, rice husk, oil palm, and flax have been used to strengthen PLA to improve its mechanical properties. Some of the mechanical properties of natural fiber-based PLA composites that have primarily been studied in terms of tensile strength, flexural, and impact strength are summarized in Table 7.

Shih et al. [65] used a melt-mixing method to create bio-composites from recycled plastic chopsticks and a PLA matrix. The tensile strength of the composites improved significantly with the fiber material, reaching 115 MPa in the composites reinforced with 40% fibers, which was roughly three times higher than that of pure PLA. The tensile strength dropped significantly when 60% of the MRDCF was reinforced with PLA. This phenomenon might be due to the high content of fibers which may subsequently influence the mechanical properties of the composites [65,167,168,169,170,171,172,173]. Islam et al. [174] found a Young’s modulus of 10.9 GPa and a tensile strength of 82.9 MPa in alkali-treated industrial hemp fiber (optimum loading of 30 wt %)-reinforced PLA composites. Interfacial shear strength (IFSS) results demonstrated that interfacial bonding was also increased by alkali treatment of fibers, leading to improved composite mechanical properties. Alkali-treated sisal fiber reinforced soy protein resin-based bio-composites were developed by Kim and Netravali [175], who found that the treatment increased fracture stress and stiffness in the sisal fiber by 12.2% and 36.2%, respectively, while fracture strain and hardness decreased. This could indicate the improvement of the interfacial adhesion strength between fibers and PLA as well as the fibers with increased transparency through swelling in which higher surface tension of the fiber could allow better PLA resin spreading on the fibers, which in turn would improve the fiber/resin interfacial adhesion. Gregorova et al. [176] observed increased Young’s modulus (3.73 ± 0.247 GPa) and decreased tensile strength (37.2 ± 2.0 MPa) for PLA/spruce wood flour (40 wt %) bio-composites with different surface treatments of the wood flour. The silane treatment was found to be the most effective in enhancing the PLA matrix’s interfacial adhesion to the spruce wood flour.

Using scanning electron microscopy, Qin et al. [177] investigated the morphological and mechanical properties of polybutyl acrylate (PBA)-modified rice straw fiber (RSF)-reinforced PLA bio-composites and discovered good interfacial adhesion between PLA and RSF as well as good RSF dispersion in the polymer. When the PBA content was increased, however, poor interfacial adhesion between PLA and RSF was observed. Tensile testing revealed that the tensile strength of the PLA/RSF bio-composites increased to 6 MPa and then rapidly decreased when the PBA content in the bio-composites surpassed 7.98 wt %. They found that adding PBA to PLA reduced the tensile strength of the bio-composites while increasing the elongation at split. Yu et al. produced surface-treated ramie fiber reinforced PLA bio-composites and discovered that PLA-based composites outperformed neat PLA in terms of tensile strength. Due to strong interfacial adhesion between the ramie fibers and the PLA matrix, the mercerization of fibers (alkali and silane) increased the tensile strength and strain of the composites, with a maximum strength of 64.24 MPa (the composite treated with alkali).

### 5.2. Thermal Properties of PLA-Based Green Composite

Thermal analysis refers to the analysis of a change in a property of a sample, which is related to an imposed temperature alteration. For composites in a variety of applications, dimensional stability is important. During the function, poor dimensional stability induces warping and other shape changes. TMA tests can be used to determine the coefficient of thermal expansion (CTE), a parameter used to assess a material’s thermal stability.

**Figure 7:**
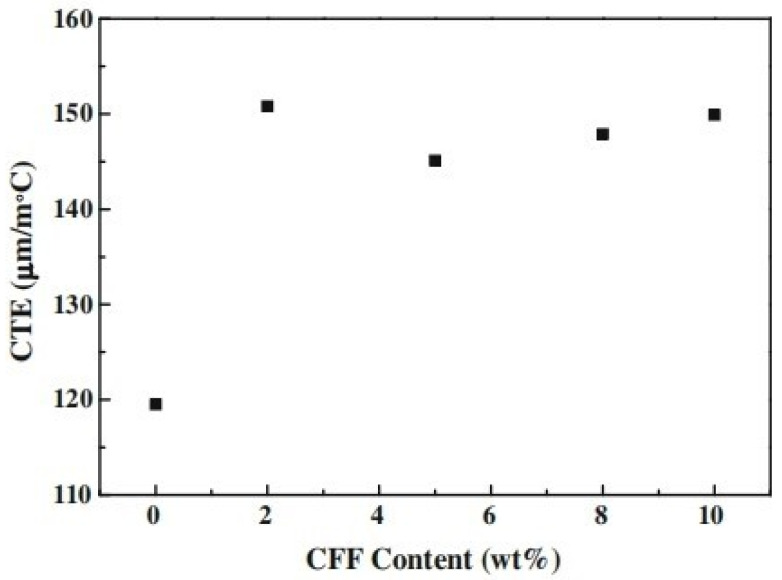
Plot of coefficient of thermal expansion for pure PLA and CFF/PLA composites with different CFF contents. (Reproduced with a copyright permission from Cheng et al. [133]).

In a study conducted by Cheng et al. [133], CTE measurements of chicken feather fiber reinforced PLA composites with different contents were measured. CTE values in CFF/PLA composites are higher than in pure PLA. This can be caused by CFF’s low thermal stability or composite defects such as interface debonding and matrix and reinforcement fracture, both of which have a major impact on the composites’ elasto-plastic behavior. The 5 wt % CFF/PLA composite has the best thermal stability among the CFF/PLA composites with different CFF materials, which is possibly due to a good distribution of CFF in the matrix. Figure 7 illustrates the plot of CTE against pure PLA and CFF/PLA composites with different content.

**Figure 8:**
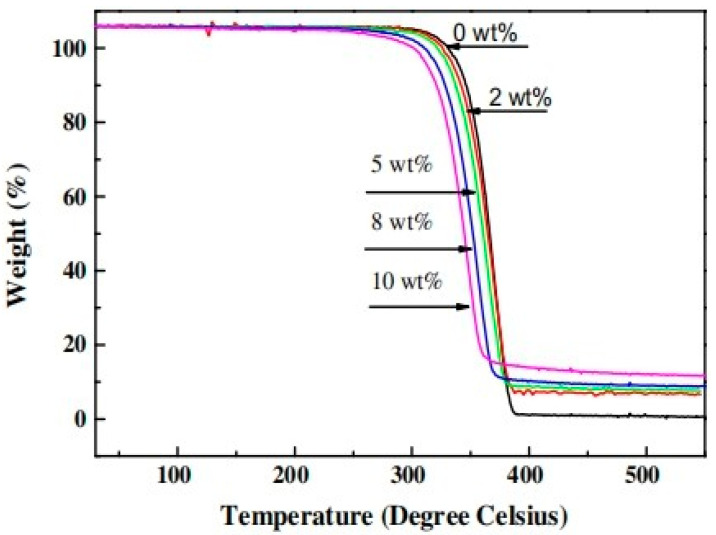
Thermogravimetric curves as a function of temperature of pure PLA and CFF/PLA composites. (Reproduced with a copyright permission from Cheng et al. [133]).

TGA was used to investigate the thermal stability of pure PLA and CFF/PLA composites. Figure 8 depicts the thermogravimetric (TG) curves of pure PLA and CFF/PLA composites as a function of temperature. CFFs degrade in three stages: (i) moisture absorbed during storage is first released from the chicken fibers; (ii) the CFF undergoes degradation from 265 to 350 °C; and (iii) the CFF begins to decompose from 350 °C onwards. The results of pure PLA and CFF/PLA composites show little difference because the curves are so similar to each other. The TG graph shows that starting at 300 °C, the weight percentage of pure PLA and CFF/PLA composites drops dramatically, primarily due to material degradation. As the materials begin to decompose at about 360 °C, a second transition occurs. CFF reinforcements improve the thermal stability of the CFF/PLA composite by acting as heat insulation barriers and preventing the permeation of volatile degradation products into the composite. With the CFF content, the weight percentage of char at 400 °C for CFF/PLA composites increases. This improvement in char formation can be attributed to CFF reinforcements’ increased heat resistance.

### 5.3. Rheological Properties of PLA-Based Green Composites

It should be noted that the fillers’ incorporation into a thermoplastic polymeric matrix typically results in the viscosity increment of the compositions [178]. This phenomenon is associated with the requirement for increased processing temperature and high injection molding speed or forming pressure, which significantly affects the material’s structure and is frequently correlated with incomplete lignocellulosic filler degradation. Generally, the addition of the particle-shaped filler increases the viscosity mentioned above while simultaneously decreasing the wall slip during capillary flow [179,180]. Numerous studies concentrate on the finished properties of composites but frequently neglect to discuss their processing and limitations due to their rheological behavior. While many researchers have focused on identifying the rheological behavior of traditional wood polymer composites based on well-defined commercial wood flour [165,166,167,168], the rheology of composites based on waste lignocellulosic materials with a variety of chemical structures and additional low-molecular products that might be emitted during melt processing is still inadequate. It should be noted that the addition of fillers typically rises the thermoplastic composites viscosity and reduces their processability [181].

According to Barczewski et al. [170], the oil content of linseed cake (LC) as an agricultural waste filler has an effect on the rheological behavior of PLA. It was demonstrated that by incorporating a suitable natural filler, self-lubricating environmentally friendly composites with improved rheological properties can be manufactured. Reduced shear stress and viscosity values were obtained for the composites as a result of increased wall slip caused by the presence of oil residue in unmodified LC. The oil from a natural waste filler demonstrated plasticizing effect on the PLA matrix by partially compensating the negative constraints imposed by the high concentration of lignocellulosic particles. The addition of up to 10 wt % of ground LC significantly improved the rheological properties of the composites, including decreased viscosity and increased wall slip. When combined with significant plasticization and reduction in the brittleness of the PLA matrix observed in Ares et al. [182], the concurrently modified rheological behavior of PLA–LC composites led to their great relevance for extruded or injected molded parts. Unfortunately, when composites contain more than 20% LC and LCA by weight, the high oil content of both types of LC fillers (pure and defatted) imposes severe limitations on plastification in a dynamic kneader chamber.

Cui et al. prepared PLA/regenerated cellulose (RC) nanocomposites comprising 0.5, 1.0, 1.5, 2.0, 3.0, and 5.0 wt % cellulose via the Pickering emulsion process [183]. The viscosity rapidly increased as the cellulose concentration increased, and yield stress occurred at 0.5 wt % RC content. Nizamuddin et al. [172] investigated the suitability of hydrochar produced by microwave-induced hydrothermal carbonization (HTC) of rice straw as a filler in PLA/HC blends. Hydrochar was found to improve the structure, chemical composition, and mechanical, thermal, and rheological properties of the PLA matrix. The storage modulus (G’) increased as the hydrochar loading increased; for instance, a higher G’ was observed at higher hydrochar loading. This behavior is a result of hydrochar clusters, which reduced the mobility of PLA chains, resulting in increased resistance to flow and, consequently, increased storage modulus. Additionally, Yang et al. [173] demonstrated that pulp fiber and wood flour addition altered the rheological behavior of the composite by increasing the viscosity of the composite in the presence of fibers and decreasing it as the test frequency increased. The absence of Newtonian behavior at low frequencies indicates a transition from liquid-like to solid-like viscoelastic behavior, indicating that the fibers addition altered the rheological behavior of the PLA composites by perturbing the normal polymer flow and impeding the mobility of the chain segments within the flow [184]. However, increasing the fiber content to 40% would further reduce fiber dispersion in the melt matrix, resulting in high-viscosity composites [185].

## 6. PLA Blend Composites

### 6.1. Mechanical Properties of PLA Blend Composites

Several models are presented in this section, along with a brief summary of each. To predict the mechanical properties of two-phase mixtures with matrix-dispersed morphology, several theoretical and empirical models have been proposed. The rule of mixtures and the so-called series model are the two basic models that describe the upper and lower bounds for several properties of composites, respectively. Each step is assumed to contribute independently to the overall modulus, proportional to its volume fraction, in the rule of mixtures model, also known as the parallel model in Equation (4):

(4) $$K_{b}=K_{m}\emptyset_{m}+K_{d}\emptyset_{d}$$

where *b*: blend; *m*: major phase; *d*: disperse phase, /: volume fraction, *K*: property.

Indeed, these models are general “mixture rules” that can be extended to a variety of properties, with K_i_ referring to the specific property. In a completely percolating network, the parallel model maximizes the contribution of the conductive process and implicitly assumes perfect interaction between particles. This model is useful in the case of continuous fiber composites in the direction parallel to fibers, but it overestimates other forms of composites significantly. The series model, on the other hand, assumes no particle interaction, so particle contributions are limited to the region of the matrix embedding the particle. As a result, Equation (5) predicts the modulus of composites with the series model.

(5) $$\frac{1}{K_{b}}=\frac{\emptyset_{m}}{K_{m}}+\frac{\emptyset_{d}}{K_{d}}$$

Maxwell solved the mechanical properties of randomly distributed and non-interacting homogeneous spheres in a homogeneous medium using potential theory (Equation (6)).

(6) $$K_{b}=K_{m}\left[\frac{K_{d}+2K_{m}+2\emptyset_{d}\left(K_{d}-K_{m}\right)}{K_{d}+2K_{m}+\emptyset_{d}\left(K_{d}-K_{m}\right)}\right]$$

where *K* stands for mechanical properties, such as modulus. Other theoretical models have attempted to explain the mechanical properties of two-phase co-continue blends. For these polymer blends, assuming the blend macroscopically homogeneous and isotropic, A relation for the tensile modulus is as follows (Equation (7)):

(7) $$E_{b}^{1/5}=E_{b}^{1/5}\emptyset_{m}+E_{b}^{1/5}\emptyset_{d}$$

Lyngaae-Jorgensen et al. discovered a relationship for the modulus of blends above the minor component’s percolation threshold (T), where *T* values are in the range of 1.7–1.9 and /*c* is 0.16 (Equation (8)).

(8) $$E_{b}=E_{m}+\left(E_{d}-E_{m}\right){\left[\frac{\emptyset_{d}-\emptyset_{c}}{1-\emptyset_{c}}\right]}^{T}$$

The cross-orthogonal skeleton (COS) model was developed by Kolarik et al. for the simultaneous prediction of modulus and yield stress of polymer pairs with co-continous morphology equation (Equation (9)):

(9) $$E_{b}=E_{m}\left(1-f^{2}\right)+E_{d}f^{2}+\frac{2f\left(1-f\right)}{\left(1-f\right)/E_{m}+f/E_{d}}$$

where *f* is related to volume fraction by *ϕ_m_* = (1 – *f*)^2^ (1 + 2*f*).

Based on Table 8, the PLA/NBR19 composite has the highest tensile strength, 51.57 MPa, and the PLA/PP composite appears to have the lowest tensile strength, 33.71 MPa. Results from dynamic mechanical analysis and rheological analysis of blends suggested better compatibility between PLA and NBR19, as reflected in the more homogenous and tougher blend, which was in good agreement with the interfacial tension data and the fine morphological structure. As for the tensile modulus, PLA/PP/Cloisite30B composite has the highest tensile modulus with 3.10 GPa, whereas PLA/PA composite has the lowest tensile modulus with 1.20 MPa. This is due to the fact that when stress is imposed on a nanocomposite, incorporated nanoparticles can act as stress concentration points, leading to the progress of crazes at the interphase and happening of the failure.

Since the flexural strength of other composites is not available, PLA/ABS/SAN-GMA composite has 62.9 MPa flexural strength, which is the highest and PLA/ABS composite has the lowest with 45.6 MPa flexural strength. The same goes for the flexural modulus, whereby PLA/ABS/SAN-GMA is the highest, with 2.30 GPa, and PLA/ABS is the lowest, with 1.96 GPa. The composite with the highest impact is PLA/PA composite, with 276 kJ/m^2^, whereas the composite with the lowest impact is PLA/PP/Cloisite30B, with 3.6 kJ/m^2^. The impact is related to the more homogeneous microstructure and apparently a better interfacial adhesion since the PA dispersed phase was covered with the matrix.

PLA bio-blends with a predominantly bio-sourced PA10.10 in the composition range 10–50 wt % were prepared by melt blending in order to overcome the brittleness of PLA. According to Cailloux et al. [189], the effect of the viscosity ratio on the PLA/PA10.10 bio blends morphology and mechanical properties revealed the expected two-phase structure independently of the formulation considered. The sea-island morphology confirms the basic immiscible nature of the PLA/PA10.10 bio blends, where the PLA/PA composite was prepared by melt blending and furthered by compression molding. PLA/PA engineered blends are promising technical progress that opens new perspectives for PLA-based products in technical application.

PLA/ABS/SAN-GMA replacement is beneficial in the cost aspect because the price of ABS is higher than that of PLA. However, the incompatibility between PLA and ABS as well as the brittleness of PLA makes it difficult to achieve the desired material properties. In the study by Jo et al. [186] on the effects of compatibilizers on the mechanical properties of ABS/PLA composites shown to increase the content of polybutadiene moiety of ABS and introduction of proper compatibilizer and heat, a stabilizer significantly enhanced the mechanical properties of ABS/PLA composites. ABS/PLA was prepared by extradition and injection molding. The addition of SAN-GMA exhibited tensile strength higher than 40 MPa, the target value to be applied for the car console boxes. The impact strength was also increased due to the addition of SAN-GMA. Composites composed of ABS and PLA were prepared to develop ABS-based automobile console boxes with improved environmental friendliness.

PLA/PP is one of the simple approaches to overcome the main problems of the PLA, which are its low impact strength and elongation at break. A study conducted by Ebadi-Dehaghani et al. [188] on experimental and theoretical analyses of mechanical properties of PP/PLA/clay nanocomposites showed that PLA/PP requires compatibilizer to achieve better tensile properties and broke upon impact.

### 6.2. Thermal Properties of PLA Blend Composites

**Figure 9:**
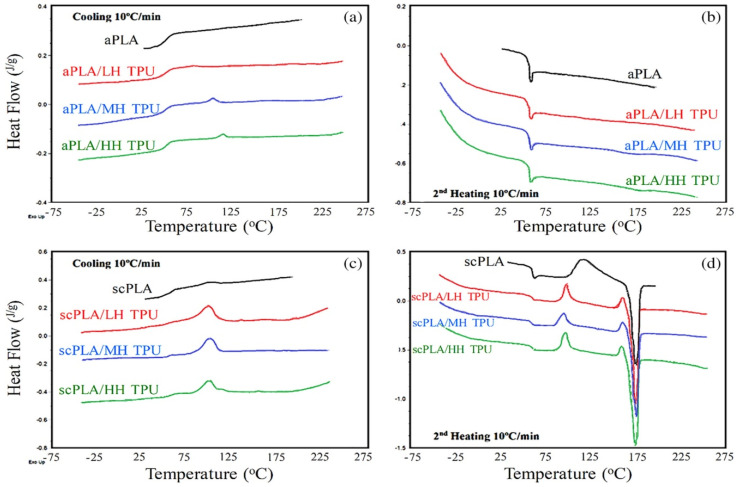
The DSC cooling and second heating thermograms of the (**a**,**b**) aPLA/TPU and (**c**,**d**) scPLA/TPU blend systems processes [Reproduced with a copyright permission from Nofar et al. [190].

DSC and DMA were used to examine the PLA/TPU blends’ crystallization and thermal behavior. The DSC cooling and second heating thermograms of amorphous PLA (aPLA)-aPLA/TPU and semicrystalline PLA (scPLA)-scPLA/TPU blends processed at 190 °C are shown in Figure 9. Since the thermograms of the aPLA/TPU blends processed at 150 °C were close to those of the aPLA/TPU blends processed at 190 °C, they were not displayed. The HS crystal melting and melt crystallization peaks are seen in the blends with aPLA, and they differ with the increase in the HS content of the TPUs. The crystal melting and melt crystallization peaks of TPUs and scPLA overlapped in blends with scPLA, making it difficult to distinguish between them. According to the second heating graph (Figure 9d), the presence of TPU dispersed phases sped up the cold crystallization of the scPLA from nearly 120 °C in the tidy scPLA to about 100 °C in the blend systems.

The crystallization temperatures in blends with aPLA increased as the TPUs with higher HS content were used, according to the recorded results in Table 9. TPU crystallization was significantly decreased to lower temperatures in blends with scPLA, well below the PLA crystallization temperature. This may mean that PLA crystallization slowed the rate of TPU HS crystallization. In the presence of the TPU dispersed process, however, the crystallization heat enthalpy of PLA, which was merged with that of TPU, was significantly increased. In scPLA blends with 15% TPU, the crystallization heat enthalpy increased from around 4.5 J/g to around 20 J/g. TPU HS crystallization could be attributed to a maximum of 3 J/g and the 15 wt % TPU material used in the blend. The PLA crystallization was accelerated by the TPU dispersed process, but the PLA crystallization was delayed by the PLA, according to the crystallization peaks. The glass transition temperatures were not significantly affected, but due to the presence of crystalline structures in the matrix, the systems with scPLA showed higher transition temperatures.

Table 9 also includes the results of DMA temperature sweep experiments on these samples. With the addition of TPU with varying HS contents, the storage modulus and tan values did not change significantly. The damping factor was decreased in the scPLA-based blends with TPUs containing higher HS contents, according to the DMA results, despite the fact that the glass transition temperatures were not affected significantly by the addition of TPU and the improvement in the HS content. This may be due to the stiffer structure and possibly better phase compatibility. The addition of 15 wt % TPU with various HS contents had no major effect on the storage modulus at 30 °C. The addition of TPU to the scPLA-based blends, on the other hand, significantly increased the storage modulus at 90 °C. When TPUs with higher HS contents were used, the increase was even greater. The modulus of the neat scPLA increased to 12.6 MPa in the scPLA/HH TPU. This may be due to the synergistic effect of improved PLA crystallization and higher HS content TPU incorporation.

### 6.3. Rheological Properties of PLA Blend Composites

By combining two or more polymers, properties such as thermal conductivity, dynamic rheology, and viscoelasticity can be tailored. Blending modifies the microstructure of a polymer, affecting its glass transition, crystallization, cross-linking, phase separation, and orientation, as well as the viscoelastic behavior of blends [191,192].

Yuhui Qiao et al. [186] investigated the fibrillated PCL/PLA composites’ morphology, rheology, isothermal crystallization, and foaming behavior. Following hot stretching, samples comprising 5%, 10%, and 20% PLA were melt-blended with PCL using a twin-screw extruder. The melt rheological behavior of the samples prior to stretching indicated that rising the amount of PLA particles increased the composites’ viscosity and storage modulus. When the PLA domains were fibrillar, this effect became more pronounced. Additionally, rheological results revealed that the fibrillated PCL/PLA composites exhibited a solid-like behavior, as indicated by a plateau in the storage modulus in the low-frequency region. Besides that, when high PLA nanofibrils are used, the interaction between a large number of induced PCL crystal nuclei and increased viscosity prevents PCL molecules from moving freely, resulting in higher activation energy of isothermal crystallization. Harada et al. [187] used a twin-screw extruder and injection molding machine to combine poly(lactic acid) (PLA) and poly(butylene succinate) (PBS) in the existence of lysine triisocyanate (LTI). The overall trend of the MFR level reduced as the amount of PBS increased, implying that PBS may also be necessary for reactive blending. When torque data were measured in an internal mixer, the viscosities of PLA and PCL cross-linked blend composites by TPP19 and DCP20 were increased.

Mofokeng et al. [188] and Jain et al. [189] investigated the dynamic mechanical performance of PLA/PCL blends with TiO_2_ and micro-clay, respectively. They observed a decrease in the storage modulus (E′) of PLA as a result of PCL’s plasticizing effect. According to Faker et al. [190], the flow characteristics and viscoelastic properties of PE/EVA blends showed a strong interfacial interaction between the phases. Singla et al. [193] evaluated the rheological properties of poly (lactic acid)/ethylene-co-vinyl-acetate copolymer (EVA) blends with EVA volume fractions ranging from 0 to 0.35. The substantial increment in the blends’ viscosities could be associated with increased interactions between matrix PLA and EVA, which was also largely supported by enhanced melt stability observed during processing via TGA analysis.

Ruyin Wang et al. [192] investigated the rheological properties and thermal stability of PLA/PBSA/POSS melt-mixed composites. In comparison to Octavinyl POSS (vPOSS), epoxycyclohexyl POSS (ePOSS) had a greater effect on improving G′, G′′, and η*, indicating that the material has a higher melt elasticity and a broader processing window after the addition of ePOSS. The solution behavior and FTIR analysis confirmed the interactions between the epoxy groups of ePOSS and the hydroxyl and carboxyl groups of PLA/PBSA, which are highly correlated to the increase in viscosity and improved dispersion of ePOSS. Stabilization increased with the ePOSS addition, which should be contributed to the reduced hydroxyl and carboxyl end groups and the resulting restricted molecular chain mobility.

## 7. PLA Hybrid Composites

Polylactic acid (PLA) and its copolymers have been extensively used in the biomedical field, specifically in orthopedic applications. However, the main problem associated with PLA-based polymers is that they have low mechanical properties, making it difficult to achieve stiffness levels equivalent to metallic implants. As a result, problems such as increased implant size, longer degradation periods, and adverse effects from acidic species often emerge. The use of a biodegradable reinforcement in the fabrication of fully absorbable composite materials will solve the problems described above as a result of numerous studies towards hybrid reinforcement-based composites for structural and aerospace applications by researchers. Hybrid composites are usually used when a combination of properties of different types of fibers wants to be achieved, or when longitudinal as well as lateral mechanical performances are required. Up until today, various PLA hybrid composites have been studied, and their mechanical and thermal properties vary in accordance with the type of fiber reinforcement.

### 7.1. Mechanical Properties of PLA Hybrid Composites

Table 10 shows mechanical properties of various PLA hybrid composites, including their tensile strength, tensile modulus, flexural strength, flexural modulus, and impact. All these data are extracted from studies by researchers of PLA hybrid composites.

From Table 10, it is observed that the highest tensile strength was achieved by PLA/banana/sisal fiber at 79.00 MPa, while PLA/hemp/sisal obtained the lowest tensile strength at 46.25 MPa. However, PLA/hemp/sisal achieved the highest tensile modulus at 6.10 GPa, followed by the lowest value of tensile modulus at 0.88 GPa, which was obtained by PLA/polycaprolactone/oil palm mesocarp.

Asaithambi et al. [194] suggested that the addition of untreated hybrid fiber to virgin PLA provided support by acting as a stress bearer for the PLA matrix. By alkali treatment towards the fiber, the adhesive characteristics of fiber surface would be improved due to the elimination of natural and artificial impurities. Pappu et al. [75] reported that fiber length/orientation, matrix properties (kinetics and crystallinity), and processing techniques all influence the properties of natural fiber reinforced PLA composites. It is also worth noting that fiber surface roughness aids mechanical interlocking with matrices.

In terms of flexural strength, PLA/banana/sisal fiber obtained the highest value at 125.00 MPa. It also can be observed that PLA/polycaprolactone/oil palm mesocarp achieved the lowest value of flexural strength at 21.45 MPa. Surprisingly, as for flexural modulus, PLA/montmorillonite nanoclay/short kenaf achieved the highest value at 7.50 GPa, while PLA/flax/jute achieved the lowest value at 2.25 GPa.

In the study conducted by Asaithambi et al. [194], the flexural properties of PLA composites improved due to the mercerization process that transforms crude cellulose structure I into a refined cellulose structure II with reactive groups, resulting in short-length crystallites capable of making intimate bonds with PLA matrix. Following alkali treatment, it increased the strength of chemical bonding between fiber and PLA matrix in composites.

### 7.2. Thermal Properties of PLA Hybrid Composites

The thermal properties of PLA are very reliant on molecular weight, molecular weight distribution, and composition. A variety of PLAs with different physical properties can be easily obtained due to its stereochemical composition and thermal history, which decided that PLA is either amorphous or semicrystalline in solid state [205]. Glass transition temperature (T_g_) is very important for amorphous PLA since the T_g_ zone is a place where polymer chain mobility occur. On the other hand, both T_g_ and melting temperature (T_m_) are important parameters for semicrystalline PLA in order to study the behavior of PLA [206].

The addition of hybrid fiber reinforcement will alter both T_g_ and T_m_ of PLA composites with a suitable ratio of fiber reinforcement to the polymer matrix. Table 11 shows a few PLA hybrid composites along with their thermal properties.

From Table 11, it can be seen that the highest T_g_ is PLA/coir/pineapple leaf fiber with a value of 290.07 °C, while the lowest T_g_ is PLA/hydroxyapatite/membrane mat with a value of 153.60 °C. Several factors can affect the value of T_g_, such as molecular adhesion, chain modification ability, steric effect, molecular weight, bonding nature, and cross-link density [213]. However, the hybrid composite that achieved the highest T_m_ was PLA/chitosan/basalt with a value of 63.32 °C, while PLA/hydroxyapatite/membrane mat achieved the lowest T_m_ with a value of 51.29 °C

To date, various studies have been conducted on coir/pineapple leaf fiber reinforced with polymer [214,215,216]. Siakeng et al. [207] investigated the thermal properties of coir and pineapple leaf fiber reinforced polylactic acid hybrid composites. Coir fiber (CF) and pineapple leaf fiber (PALF) were chosen as fiber reinforcement, and the hybrid composite was fabricated using a melt blending process. Thermal degradation of pure PLA was found to be faster with the addition of CF and slower with the addition of PALF reinforcement. Subsequently, when PALF was combined with PLA as a reinforcing fiber, it performed better compared to CF in terms of improved thermal stability.

### 7.3. Rheological Properties of PLA Hybrid Composites

Melt flow behavior of PLA hybrid composites is deduced by the MFI test, which provides practical and specific information about changes in rheology viscosity of polymer melt and the processability of composite materials.

**Figure 10:**
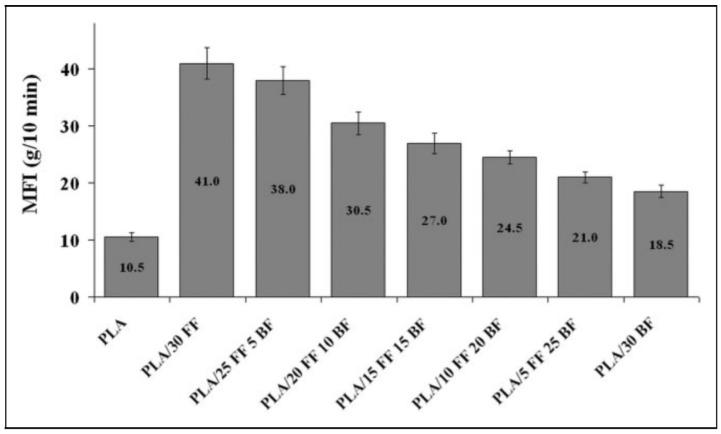
MFI values of PLA and composites. (Copyright permission from Najah Eselini et al. [217].) MFI: melt flow index; PLA: poly (lactic acid); BF: basalt fiber; FF: flax fiber.

Najah Eselini et al. [217] conducted a study on the rheology properties of basalt fiber and flax fiber reinforced PLA hybrid biocomposite. The result shows that both FF and BF reinforced PLA matrices rise up the higher MFI values, as shown in Figure 10. Individual additions of FF increased the melt flow rate of pristine PLA by approximately fourfold. On the other hand, the addition of BF resulted in a decrease in the MFI values of hybrid composites, with the lowest MFI observed for the hybrid containing the most BF. The MFI value of the PLA/30 BF composite was lower than that of all hybrid forms and slightly higher than that of unfilled PLA. The addition of more fiber to PLA results in an increase in shear during composite processing. This could explain the increase in the MFI value of PLA following fiber loading. Numerous studies in the literature have indicated that natural fiber-reinforced composites exhibit shear thinning behavior [218,219].

Boubkeur et al. [220] conducted research on organomontmorillonite (OMt)/graphene (Gr)–PLA/PCL hybrid nanocomposites using the melt compounding technique. The addition of OMt/Gr mixtures alters the rheological properties of a pure PLA/PCL binary blend significantly [221]. By adding organoclays, low-frequency rheological properties (loss modulus, storage modulus, and complex viscosity) were increased, and this effect was greatly improved when the OMt was linked with epoxy functionalized graphene. Additionally, polymer chains that have been confined by nanofillers may demonstrate a constraint in their mobility. This phenomenon has been discussed for the PLA matrix filled with OMt/graphene mixtures [222].

Huda et al. [223] studied the effect of silane-treated and untreated talc on the rheology properties of poly(lactic acid)/newspaper fibers/talc hybrid composites. The obtained result indicates that the complex viscosity declined with increasing frequency in both PLA-based composites. PLA’s low viscosity may contribute to interface polymer aggregation. In general, when fibers interact, the viscosity decreases, and thus, the generation of viscous heat decreases as well, resulting in a lower melt temperature. Davachi et al. [223] investigated the rheological behavior of novel antibacterial hybrid nanocomposites based on PLLA/triclosan/nano-hydroxyapatite (nHA). They discovered that the addition of nHA increased all rheological parameters, but no G′ and G′′ intersection was detected at lower frequencies.

Poly(lactic acid)/sesbania gum/nano-TiO_2_ composites were prepared by melt blending in the mixing chamber of a torque rheometer, performed by Qing Zhang [221]. The result indicates that as the amount of nano-TiO_2_ increased, both the feedstock and the plasticizing peaks exhibited an increased tendency. The melt viscosity of the nanocomposite increased, and thus the torque value increased as well. Rostami et al. [224] investigated the rheological characteristics, and MWCNT-reinforced PLA biodegradable hybrid nanocomposites. The result indicated that incorporating fGnPs and fCNTs simultaneously increased the elasticity and viscosity of the samples, which are the characteristics of nanostructured materials. The nonterminal behavior in storage modulus and viscosity upturn at low frequencies became more pronounced as the fCNT content increased. This denoted the three-dimensional networks formation between single particles (particle-particle) and/or the particles and the matrix (particle-matrix). By increasing the dispersion degree and concentration of nanofillers, the strength of these networks or the extent to which they form can be increased [224,225,226,227].

## 8. Applications of PLA-Based Green Composites

This section summarizes the applications of PLA and delves into the relationship between PLA’s mechanical/physical properties and the necessary features for various applications, as well as highlighting current problems that need to be investigated further in order to improve PLA performance.

### 8.1. Wound Management and Stent Applications

PLA and its copolymers have been used in a variety of wound care applications, including surgical sutures, wound healing after dental extractions, and avoiding postoperative adhesions. The rate of PLA degradation in sutures has recently been shown to be dependent on the magnitude of the applied stress. Due to viscoplastic flow causing creep rupture or fatigue failure, PLA may exhibit premature failure at stress magnitudes that are substantially lower than the yield strength and the ultimate tensile strength of the content [65]. Consequently, system failure can occur in some applications well before the material is expected to fail due to degradation in vivo. During the crimp and expansion phases, a bioresorbable PLA stent, for example, is subjected to extremely high stresses and strains. The stent usually has significant viscoplastic residual strains after deployment. It must also preserve mechanical integrity when subjected to a high number of load cycles with small stress and strain amplitudes [228]. By altering the mechanical reaction of the devices to their in vivo conditions, the use of degradable, non-linear, viscoplastic materials poses many new challenges to the production and use of bioresorbable stents. As a result, PLLA is the most widely used substrate for bioresorbable scaffolds/stents. Due to the lower stiffness and strength of PLLA relative to metals, the struts must normally be thicker than those used in traditional metal stents to achieve the desired radial strength [229]. Poor deliverability, platelet deposition, and vessel injury can result. To resolve these issues, a thorough understanding of the mechanical response of the PLA implant/device is needed. This necessitates a thorough understanding of how the material’s strength and stiffness shift over time as a result of time-dependent microstructural processes, how the material responds during deterioration, and how the material undergoes non-linear viscoplastic deformations at finite deformations [228]. Other uses, such as ligament and tendon repair, as well as urological surgery, require a longer retention of strength, and PLLA fibers are the preferred material.

### 8.2. Drug Delivery System-Based PLA

PLAs have been used to provide continuous drug release for a range of medicinal agents, including contraception, narcotic antagonists, local anesthetics, vaccines, peptides, and proteins, for extended periods of time. Erosion, diffusion, and swelling are three ways that polymeric drugs can be re-released. The breakage of ester bonds in PLA occurred at random due to hydrolytic ester cleavage, resulting in system erosion [230]. The hydrolytic products of this form of decomposition are then converted into non-toxic subproducts that are excreted by normal cellular activity and urine. Many medications, including psychotic, restenosis, hormones, oridonin, dermatotherapy, and protein, were encapsulated using PLA and its copolymers in the form of micro- or nanoparticles (BSA). PLA particles were made using solvent evaporation technology and were found to be excellent candidates for drug delivery system design [91]. The mechanical stability and crystallinity degree of PLA in the formulation can be used to tune the challenge of managed drug release.

### 8.3. Orthopedic and Fixation Devices

The difficulties that come with using PLA in orthopedics are highly dependent on the position and form of the system that will be used. While most devices are made of steel or titanium to ensure that they can withstand in vivo loading for an extended period of time, polymers have an advantage over metal implants in that they pass stress to the damaged area over time, enabling the tissues to heal [68]. Another significant benefit is the avoidance of a second surgical operation to remove unnecessary hardware, which lowers medical costs and allows for incremental tissue function regeneration as the implant degrades naturally without the use of enzymes or catalysts [231]. PLA has previously been used to make biodegradable screws, fixation pins, plates, and suture anchors. These absorbable screws and pins have become increasingly popular in clinical practice, particularly in situations where high mechanical stiffness or strength is not needed. The knee, shoulder, foot and ankle, hand, wrist, elbow, pelvis, and zygomatic fractures are all important orthopedic areas. In other cases, high-performance PLA is needed, which has proven to be difficult to achieve. Controlling the L/D ratio in the polymer improved the mechanical properties of PLA, with the ratio of L/D 85/15 being polymerized and the prepared PLA being used to produce screws and fixation plates for fracture fixation [68]. The findings revealed that the plates could be used to repair fractures without the need for external help. In contrast to their metallic counterparts, these degradable devices were found to have comparable success rates. However, in these applications, it is critical that the applied stress never exceeds the implant’s ability, causing permanent deformation or premature failure due to viscoplastic flow or fracture.

### 8.4. Tissue Engineering and Regenerative Medicine

PLA consumption has increased exponentially in tissue engineering, one of the most exciting interdisciplinary and multidisciplinary research fields. In tissue engineering and regeneration, scaffold materials and fabrication technologies are critical [232]. PLA matrix materials have sparked a lot of interest as transplantation supports because they fade away from the transplantation site over time, leaving a perfect patch of natural neo tissue behind. PLA has been studied for tissue engineering applications, such as bone scaffolds, due to its excellent biocompatibility. However, the existence of various tissues necessitates a material with a pre-determined biodegradation profile. PLA’s mechanical properties for tissue engineering have been stated to be improved using a variety of methods, including blending, composites formation, and co-polymerization [70]. Three-dimensional porous PLA scaffolds for culturing various cell types have been developed for use in cell-based gene therapy for cardiovascular disorders, muscle tissue regeneration, bone and cartilage regeneration, and other treatments for cardiovascular, neurological, and orthopedic conditions [233]. In two other experiments, osteogenic stem cells were seeded on scaffolds made of this material and inserted in bone defects or subcutaneously to mimic both endochondral and intramembranous ossification processes in bone formation. Because of the high strength of PLLA mesh, 3D structures such as trays and cages can be developed. The PLA can take anywhere from 10 months to 4 years to degrade, depending on microstructural factors including chemical composition, porosity, and crystallinity, all of which can affect tensile strength for particular applications. Furthermore, isolated cells can be stimulated to regenerate tissues and release drugs such as painkillers, anti-inflammatories, and antibiotics, which has recently prompted their investigation as cell transplantation scaffolds.

### 8.5. Components in Electrical Towers

PLA has been known due to its tremendous mechanical strength and stiffness to be applied in structural applications. In a recent review, Asyraf et al. [54] suggested that PLA has a high potential to replace unsaturated polyester resin in the fabrication of pultruded cross arms. In this manner, the tensile strength and modulus of PLA is higher as compared to polyester, which are around 49.6 MPa and 3.60 GPa, respectively [79]. The PLA polymer also is expected to have good creep, quasi-static bending and electrical resistance properties, which are suitable for civil and energy engineering applications [110,234]. The PLA seems to be suitable for cross arm building materials. This article also suggested that the PLA can be reinforced with flax fiber to produce cross arm in order to replace glass fiber reinforced unsaturated polyester composites.

### 8.6. Automotive

PLA is a well-known polymer in composites for its mechanical strength, stiffness, and chemical resistance. Jae-won et al. [235] used jute fiber as reinforcements in PLA polymer to enhance the heat resistance and impact strength of the composites. They found out that the alkali-treated of 10 wt % jute fiber exhibited remarkable tensile and flexural strength, which were 55 MPa and 108 MPa, respectively, as compared to without plasma treatment. The alkali-treated jute/PLA composites displayed good interfacial adhesion between fiber and matrix. This research also implemented post-treatment, which is an annealing treatment after the composites are fabricated. They discovered that the treated green composites had a remarkable increase in heat resistance. In the end, the PLA composites are potentially suitable for automotive parts such as indoor panels, engine cover, car spoiler, engine mounting rubber, and antiroll bar [236,237,238,239,240]. In order to implement PLA composites as automotive components, they should be well-designed using concurrent engineering techniques and finite element analysis in order to achieve optimum function ability and product property [241,242,243,244,245].

### 8.7. Packaging

PLA is a famous biopolymer usually implemented in food packaging applications. In general, there are several efforts that can be used to enhance PLA properties for extending its applications in the packaging sector. Currently, the melt blending technique is one of the processes that sparks significant interest among researchers due to easy, cost-effective, and readily available processing technologies at the industrial scale [187]. From this point of view, PLA is suggested to be used with poly(hydroxybutyrate) (PHB) because of its similar melting temperature and high crystallinity, which could present as a good candidate to blend with PLA [246]. From the physical–mechanical properties, it can be deduced that the PHB functions as a nucleating agent in PLA, which enhances the overall mechanical resistance and water barrier behaviors. PLA–PHB mixtures are good candidates in the controlled release of active compounds in the progress of active packaging systems. PLA–PHB blends are demonstrated as highly promising candidates for substituting petroleum-based polymers currently used for food packaging.

## 9. Challenges and Opportunities

PLA is a leading biopolymer for a variety of applications, and the ability to alter its properties for specific applications has catalyzed extensive research aimed at utilizing these materials in novel ways and applications. PLA-based green composites have also been the subject of much study and development in recent decades. PLA-based composites using natural fiber or cellulosic materials, as well as PLA blend and PLA hybrid composites, are discussed in this paper. PLA-based green composites are currently comparable with other synthetic polymer composites in terms of mechanical and thermal properties, and tensile and flexural strength values are approaching synthetic standards. Green composites based on PLA are also utilized in a variety of industries, including packaging, disposable waste, structural material, and medical applications. The compatibility of the hydrophobic PLA matrix and the hydrophilic natural fibers has been the most challenging problem in producing PLA-based composites from natural fiber, resulting in nonuniform fiber dispersion within the matrix and poor mechanical characteristics. As a result, additives such as chemical coupling agents or compatibilizers, as well as treatment of hybridization with stronger materials, may be suitable options for improving the internal bonding and adhesion between fibers and PLA in composite manufacturing. More research is required to expand their application range, which includes enhancing PLA’s brittle characteristics so that it may be used in applications that need plastic deformation at greater stress levels. Modifiers can be utilized to enhance stiffness at high temperatures and increase the degradation rate of PLA, which has a low glass transition temperature and poor thermal stability. To assist the future manufacturing of PLA-based composites, when using natural fiber for suitable applications, fundamental research on variables linked to strength must be performed. Greater knowledge of the molecular structure and interfacial interaction between the matrix and natural fibers, as well as the connection between structure and property, would be a significant advancement in this field of study. PLA offers several advantages for the future, and with increasing oil costs, corn-based plastic has economic advantages, as well. Despite all of these advantages, PLA’s low melting point compared to polymers such as PET means it has yet to be adopted for a wide range of applications.

## 10. Conclusions

PLA is a leading candidate for consumer and biomedical applications, and the ability to adapt its mechanical, physical, microstructural, chemical, and degradation properties for particular applications has catalyzed a large and increasing amount of research aimed at using these materials in new ways and applications. Our vision for PLA’s future is based on the rational assumption that no single material can meet all design criteria in all applications. As a result, future advances are likely to include PLA blends, copolymers, and impact-modified products, expanding the range of applications for this particular polymer. Aside from that, we anticipate increased research interest in PLA blends stability and composite fiber mixtures in the near future.

Several PLA blends based on synthetic or natural components have recently been found to be very effective in improving PLA properties. However, very little attention has been paid to studying blend stability under various aging conditions (e.g., in various environments, during storage, or during reprocessing). This part is critical, and more attention should be paid to the unbiased evaluation of these new compositions’ advantages in terms of durability and applicability, as compared to previously reported compositions (PLA: PGA, PLA:PCL, etc.).

Fiber mixture composites incorporate the positive properties of various fibers. We can design the composite properties by adding seed fibers with high elongations for improved impact or stem fiber for improved stiffness by learning from nature what the role of a fiber in a plant is. Future research on PLA composites should pay more attention to the function of the reinforcing fiber. It is possible to design composite properties by experimenting with different fiber characteristics. So far, the findings have shown that the investigated composites, with their various properties, can be used for a variety of technological applications, each with its own set of requirements.

## Figures and Tables

**Table 1:** Chemical composition (wt %) of some green fibers.

Fiber	Cellulose	Hemicellulose	Lignin	Reference
Abaca	62.5	21	12	[44]
Bagasse	37	21	22	[44]
Banana	62.5	12.5	7.5	[44]
Bamboo	34.5	20.5	26	[44]
Coir	36–43	0.15–0.25	41–45	[45]
Cotton	93	3	0	[46]
Flax	71–78.5	18.6–20.6	2.2	[47]
Hemp	70.2–74.4	17.9–22.4	3.7–5.7	[48]
Jute	61–72	13.6–20.4	12–13	[49]
Kenaf	37–49	18–24	15–21	[50]
PALF	68.5	18.8	6.04	[51]
Ramie	68.6–76.2	13.1–16.7	0.6–0.7	[52]

**Table 2:** Mechanical properties of green fibers [35,53,54,55,56,57,58,59].

Fiber	Density (g/cm^3^)	Specific Modulus	Tensile Strength (MPa)	Young’s Modulus (GPa)	Elongation at Break (%)
Flax	1.5	50	345–1100	27.6	2.7–3.2
Pineapple	1.53	40	170	1.44	14.5
Hemp	1.4	50	550–900	70	1.6
Jute	1.3–1.45	38	393–773	13–26.5	1.16–1.5
Ramie	1.0	-	400–938	61.4–128	1.2–3.8
Sisal	1.45	22	468–640	9.4–22	3–7
Abaca	1.5	-	857	41	1.10
Cotton	1.5–1.6	-	287–800	5.5–12.6	7–8
Coir	1.15	-	131–175	4–6	15–40
E-glass	2.6	-	1800–2700	73	2.5
Kevlar	1.4	-	2758	62	2.5–3.7
Carbon	1.8	-	3500–5000	260	1.4–1.8

**Table 3:** General physical and optical properties of commercial amorphous PLA [82,96,97,98].

Characteristics	Unit	Amount
Physical		
Molecular weight	g/mol	66,000
Specific gravity	-	1.27
Solid density	g/cm^3^	1.252
Melt density	g/cm^3^	1.073
T_g_	°C	55
T_m_	°C	165
Specific heat (Cp)	J/kg °C	
190 °C		2060
100 °C		1955
55 °C		1590
Thermal conductivity	W/m °C	
190 °C		0.195
109 °C		0.197
48 °C		0.111
Optical		
UV light transmission		
190 to 220 nm		<5%
225 to 250 nm		85%
>300 nm		95%
Visible light transmission		95%

**Table 5:** Primary transition temperature of selected PLA copolymers [96].

Copolymer Ratio	Glass Transition Temperature (°C)	Melting Temperature (°C)
100/0 (L/D, L)-PLA	63	178
95/5 (L/D, L)-PLA	59	164
90/10 (L/D, L)-PLA	56	150
85/15 (L/D, L)-PLA	56	140
80/20 (L/D, L)-PLA	56	125

**Table 7:** Mechanical properties of PLA-reinforced natural fibers.

Fibers	Processing Technique	Mechanical Properties					References
		Tensile Strength (MPa)	Tensile Modulus (GPa)	Flexural Strength (MPa)	Flexural Modulus (GPa)	Impact (kJ/m^2^)	
Chicken feather	Extrusion + injection molding	55	4.2	-	-	-	[133]
Ramie	Hot pressing	52	-	105	-	-	[137]
Cotton	Compression molding	39.2–43.2	3.607–4.877	-	-	24.3–33.1	[7]
Kenaf (70%)	Hot pressing	223	32	254	35.5	8.2–10.8	[131]
Sugar beet pulp (10 wt %)	Compression molding	37.0–38.0	1.0035–1.0825	-	-	-	[156]
Silk (5 wt %)	Injection molding	62	4.2	-	-	-	[157]
Micro-fibrillated cellulose (10 wt %)	Direct mixing + compression	75	4.7	-	-	-	[158]
Wood flour (30 wt %)	Injection molding	56.45–60.11	5.68–6.76	-	-	35.96	[159]
Flax (30 wt %)	Solution casting + hot pressing	21	0.137	-	-	9.58–12.68	[160]
Bamboo flour	Injection molding	50	-	-	-	-	[161]
Chopped recycled newspaper cellulose fiber	Injection molding	67.4–68.4	4.9–5.7	104.4–108	5.4	23.1–23.9	[162]
Coconut	Extrusion + compression molding	64.24–71.74	2.22–2.52	101.6–104.2	-	80.14–82.6	[163]
Cordenka	Injection molding	108	4.2	-	-	8.5	[74]
Abaca	Injection molding	74	5.85	124	6.51	5.3	[164]
Man-made cellulose	Injection molding	92	8.032	152	7.89	7.9	[164]
Corn stover + wheat straw + soy stalk	Extrusion + Injection molding	58	5.55	80	6.9	23	[165]
Sisal	Injection molding	23–23.6	3.43–3.57	-	-	3.25	[166]

**Table 8:** Mechanical properties of PLA blend composites.

Polymers	Fibers	Processing Technique	Mechanical Properties					References
			Tensile Strength (MPa)	Tensile Modulus (GPa)	Flexural Strength (MPa)	Flexural Modulus (GPa)	Impact (kJ/m^2^)	
PLA/ABS	-	Injection molding	37.3	-	45.6	1.96	-	[186]
PLA/ABS/SAN-GMA	-	Injection molding	50.9	-	62.9	2.30	-	[186]
PLA/NBR19	-	Melt blending	49.63–51.57	2.65–3.15	-	-	-	[187]
PLA/NBR33	-	Melt blending	47.62–50.44	2.51–2.97	-	-	-	[187]
PLA/NBR51	-	Melt blending	44.74–49.92	2.71–3.23	-	-	-	[187]
PLA/PP	-	Melt blending	33.71–35.09	1.93–2.03	-	-	7.8–8.6	[188]
PLA/PP/PTW	-	Melt blending	37.53–38.27	2.20–2.50	-	-	32.8–34.6	[188]
PLA/PP	Cloisite 30B nanocomposites	Melt blending	36.94–40.66	2.85–3.10	-	-	3.4–3.6	[188]
PLA/PP/PTW	Cloisite 30B nanocomposites	Melt blending	39.15–39.45	2.50–2.61	-	-	4.3–4.9	[188]
PLA/PA	-	Melt blending	47.0–49.0	1.20–1.40	-	-	166–276	[189]

**Table 9:** DSC cooling and DMA temperature sweep graphs results [190].

Conditions	DSC			DMA			
	Cooling (5 °C/min)			tan δ			
	Tc (°C)	Crystallization Heat Enthalpy (J/g)	Tg, PLA (°C)	Tg (°C)	Damping Factor (Energy Dissipation)	Storage Modulus at 30 °C (MPa)	Storage Modulus at 90 °C (MPa)
aPLA	-	-	51.2	72.2	0.084	1447	2.6
aPLA/LH TPU (150 °C)	84.1	0.6	50.8	71.4	0.069	1346	2.6
aPLA/MH TPU (150 °C)	103.3	1.8	50.8	71.8	0.060	1288	4.1
aPLA/HH TPU (150 °C)	116.5	1.6	51.4	71.1	0.075	1231	3.4
aPLA/LH TPU (190 °C)	82.5	1.2	51.5	71.1	0.063	1250	2.9
aPLA/MH TPU (190 °C)	104.0	2.1	50.4	71.1	0.068	1431	3.4
aPLA/HH TPU (190 °C)	115.1	2.5	51.5	71.5	0.063	1203	3.8
scPLA	102.0	4.5	56.5	75.9	0.080	1510	3.5
scPLA/LH TPU (190 °C)	98.2	21.8	54.2	75.2	0.075	1238	4.9
scPLA/MH TPU (190 °C)	99.7	20.7	54.9	75.7	0.065	1254	6.8
scPLA/HH TPU (190 °C)	99.1	20.0	54.6	74.5	0.058	1308	12.6

**Table 10:** Mechanical properties of PLA hybrid composites.

Polymers	Fibers	Processing Technique	Mechanical Properties				Impact	References
			Tensile Strength (MPa)	Tensile modulus (GPa)	Flexural Strength (MPa)	Flexural Modulus (GPa)		
PLA	Banana/Sisal Fiber	Injection molding	79.00	4.10	125.00	5.60	47.80 kJ/m^2^	[194]
PLA	Flax/Jute	Compression molding	49.35	2.80	80.50	2.25	61.46 J/m	[195]
PLA	Polycaprolactone/Oil Palm Mesocarp	Melt blending	33.48	0.88	21.45	2.43	95.44 J/m	[196]
PLA	Montmorillonite nanoclay/short kenaf	Double extrusion	37.00	2.80	50.00	7.50	82.00 kJ/m^2^	[197]
PLA	Corn stover/wheat straw/soy stalks	Extrusion + injection molding	58.00	5.55	80.00	6.90	23 J/m	[165,198]
PLA	Hemp/Sisal	Injection molding	46.25	6.10	94.83	6.04	10.29 kJ/m^2^	[75]
PLA	Coir/Pineapple leaf	Melt mixing	18.00	5.00	33.00	5.00	4.3 kJ/m^2^	[199]
PLA	Banana/Kenaf	Molding	50.00	-	61.00	-	16.00 kJ/m^2^	[200]
PLA	Cotton gin waste/flax	Extrusion + melt blending	-	-	13.99	3.97	-	[201]
PLA	Bamboo/microfibrillated cellulose	Milling	-	4.81	53.80	-	-	[202]
PLA	Hemp/yarn	Compression molding/prepreg	62.00	6.50	122.00	9.00	25.00 kJ/m^2^	[203]
PLA	MMT clay/aloe vera	Extrusion	56.00	3.20	100.00	6.70	55.00 kJ/m^2^	[204]
PLA	Softwood flour/cellulose	Injection molding	70.00	56.00	-	-	-	[164]

**Table 11:** Thermal properties of PLA hybrid composites.

Polymers	Fibers	Processing Technique	Thermal Properties		References
			T_m_ (°C)	T_g_ (°C)	
PLA	Coir/Pineapple leaf fiber	Melt blending	290.07	-	[207]
PLA	PBSA/Starch	Extrusion	165.35	54.01	[208]
PLA	Clay/RCF	Mold blending	176.30	-	[209]
PLA	Hydroxyapatite/Membrane mat	Air jet spinning	153.60	51.29	[210]
PLA	Graphene oxide/CNT	Solution casting	154.00	57.70	[211]
PLA	Chitosan/Basalt	Reactive blending + injection molding	158.01	63.32	[212]
